# Pain and gain of auditory intrusions with video game content: Game transfer phenomena in clinical cases

**DOI:** 10.1192/j.eurpsy.2021.1705

**Published:** 2021-08-13

**Authors:** A. Ortiz De Gortari, A. Basche

**Affiliations:** 1 Psychology, University of Bergen, Bergen, Norway; 2 Resetfromtech.com, Private Practice, Santa Barbara, United States of America

**Keywords:** Game Transfer Phenomena, Gaming disorder, Hallucinations, differential diagnosis

## Abstract

**Introduction:**

Studies about Game Transfer Phenomena (GTP) have demonstrated lingering effects of playing video games manifesting as sensory, cognitive, and motoric intrusions (e.g., seeing images or hearing voices from the game after playing), transient changes in perception and self-agency. GTP are common among non-clinical players, though those with mental disorders are more susceptible. Gamers tend to appraise GTP as pleasant. Distress has been reported when GTP are experienced frequently and with specific content.

**Objectives:**

To show the interplay between GTP and patients’ symptomatology and the benefits of using the GTP framework in clinical contexts.

**Methods:**

GTP were assessed via clinical interviews and with a validated GTP scale (three cases, males, 10-16 years old, playing time 6-10 h/day).

**Results:**

The cases were characterised by i) incorporation of videogame content into hallucinations and delusions, ii) identification with a videogame character and subsequent distress provoked by hearing the character’s voice and iii) self-induced GTP as self-soothing behaviour when reducing playing time. Main GTP manifestations were in the auditory modality as sounds or voices. The primary clinical diagnoses were gaming disorder, depressive disorder, and psychosis.

**Conclusions:**

On one hand, GTP can be pleasurable and a way to cope with withdrawal symptoms from gaming disorder, though it can lead to compulsive behaviours and dissociation. On the other hand, GTP can be interpreted negatively and fulfil delusions that provoke distress and compromise mental stability. The cases reveal that the GTP framework can be an effective psycho-pedagogic method and support differential diagnosis.

**Disclosure:**

No significant relationships.

